# Left Ventricular Structure and Function in Elite Swimmers and Runners

**DOI:** 10.3389/fphys.2018.01700

**Published:** 2018-11-28

**Authors:** Katharine D. Currie, Alexandra M. Coates, Joshua T. Slysz, Rachel L. Aubry, Alanna K. Whinton, Margo L. Mountjoy, Philip J. Millar, Jamie F. Burr

**Affiliations:** ^1^Terry Kavanagh Heart Health Lab, Faculty of Kinesiology & Physical Education, University of Toronto, Toronto, ON, Canada; ^2^Department of Kinesiology, Michigan State University, East Lansing, MI, United States; ^3^Human Performance and Health Research Laboratory, University of Guelph, Guelph, ON, Canada; ^4^Fédération Internationale de Natation, Lausanne, Switzerland; ^5^Department of Human Health and Nutritional Sciences, University of Guelph, Guelph, ON, Canada; ^6^Toronto General Hospital Research Institute, Toronto, ON, Canada

**Keywords:** aerobic exercise, athletes, cardiovascular, diastolic function, echocardiography

## Abstract

Sport-specific differences in the left ventricle (LV) of land-based athletes have been observed; however, comparisons to water-based athletes are sparse. The purpose of this study was to examine differences in LV structure and function in elite swimmers and runners. Sixteen elite swimmers [23 (2) years, 81% male, 69% white] and 16 age, sex, and race matched elite runners participated in the study. All athletes underwent resting echocardiography and indices of LV dimension, global LV systolic and diastolic function, and LV mechanics were determined. All results are presented as swimmers vs. runners. Early diastolic function was lower in swimmers including peak early transmitral filling velocity [76 (13) vs. 87 (11) cm ⋅ s^-1^, *p* = 0.02], mean mitral annular peak early velocity [16 (2) vs. 18 (2) cm ⋅ s^-1^, *p* = 0.01], and the ratio of peak early to late transmitral filling velocity [2.68 (0.59) vs. 3.29 (0.72), *p* = 0.005]. The diastolic mechanics index of time to peak untwisting rate also occurred later in diastole in swimmers [12 (10)% diastole vs. 5 (4)% diastole, *p* = 0.01]. Cardiac output was larger in swimmers [5.8 (1.5) vs. 4.7 (1.2) L ⋅ min^-1^, *p* = 0.04], which was attributed to their higher heart rates [56 (6) vs. 49 (6) bpm, *p* < 0.001] given stroke volumes were similar between groups. All other indices of LV systolic function and dimensions were similar between groups. Our findings suggest enhanced early diastolic function in elite runners relative to swimmers, which may be attributed to faster LV untwisting.

## Introduction

Exercise-induced cardiac remodeling is well documented with the degree of adaptation dependent on the type of training (i.e., aerobic vs. resistance-based training) (Pluim et al., 2000; Spence et al., 2011; Utomi et al., 2013). There is also growing recognition that within aerobic or resistance-based training, different sport modalities may elicit different left ventricular (LV) adaptations (Spirito et al., 1994; Venckunas et al., 2008a; Wasfy et al., 2015). Within aerobic-based sports, the type of LV adaptation may depend on the degree of isotonic (i.e., dynamic) and isometric (i.e., static) components involved in the sport. Comparisons of land-based sport modalities have observed differences in LV structure and diastolic function between distance running (i.e., high isotonic and low isometric) and rowing (i.e., high isotonic and high isometric) (Wasfy et al., 2015). Sport-specific differences in LV mechanics have also been described, including peak untwisting velocity which contributes to diastolic filling (Beaumont et al., 2017).

Swimming is a sport that provides a unique physiological stimulus distinct from land-based exercise modalities. It would be classified as having a high isotonic component (i.e., similar to running or rowing), but moderate isometric component which falls somewhere on the spectrum between the other two sports (Mitchell et al., 2005). Unique aspects of this exercise modality which may influence exercise-induced LV remodeling include immersion of the body in water (and the potential influence of hydrostatic pressure and water temperature), a supine posture, use of both the upper and lower limbs, and a novel requirement of breath holding (Holmer et al., 1974; Holmer, 1979; Ferrigno et al., 1986). Posture specific cardiovascular adaptations have been observed following supine and upright cycle training (Ray et al., 1990); and LV differences between rowers and runners also suggest combined upper and lower limb exercise may provide a different stimulus than lower-limb exercise alone (Wasfy et al., 2015). There is limited evidence available demonstrating similar global LV systolic and diastolic function in swimmers and other land-based athletes (i.e., runners) (Colan et al., 1987; Venckunas et al., 2008a), and no studies have examined LV mechanics, which provides more detailed information on LV systolic and diastolic function. Therefore, the purpose of this study was to extend our understanding of the extent of exercise-induced cardiac remodeling with swimming through a characterization and comparison of LV mechanics, dimensions, and global systolic and diastolic function between elite swimmers and runners. Any differences in global LV function would likely also be demonstrated through differences in LV mechanics. Based on previous evidence, however, we hypothesized global LV function would be similar between groups, and therefore LV mechanics would also be similar.

## Materials and Methods

### Athletes

Inclusion criteria included males and females, 18 years of age or older, who were elite swimmers or runners, which was defined as an athlete currently competing at the Olympic or International level, or specifically identified by their national sporting group to represent their country. Swimmers were recruited from on-site advertisements and word of mouth from the 2016 FINA World Championships (25 m) in Windsor, Canada, and tested upon completion of all competitions. Runners were similarly recruited during competition season from an elite local club and tested at the University of Guelph in Canada. Sixteen athletes were included in each group, and they were matched based on age, sex, and race. Exclusion criteria included physician diagnosed cardiovascular disease, evidence of cardiovascular abnormality from a preceding 12-lead electrocardiogram (or follow-up diagnostic echocardiogram), or any language or cognitive barrier that prevented them from following English instructions. This study was carried out in accordance with the recommendations of the University of Guelph Natural, Physical and Engineering Sciences Research Ethics Board with written informed consent from all subjects. All subjects gave written informed consent in accordance with the Declaration of Helsinki. The protocol was approved by the University of Guelph Natural, Physical and Engineering Sciences Research Ethics Board (16OC027). All athletes provided written informed consent prior to participation and were asked to be at least 2 h fasted and 12 h post-exercise. Individuals completed a questionnaire that addressed their medical history and training history including their duration of competitive training, their average training volume, and their sporting event. With shoes removed, height was measured at end-inspiration to the nearest half centimeter using a stadiometer (SECA; Hanover, MD, United States), while weight was measured using an electronic scale (Tanita TBF-300 WA; Tanita, Arlington Heights, IL, United States). Body mass index and body surface area using the Du Bois and Du Bois (1989) formula were calculated. Athletes were then asked to rest in a supine position for 5 min, after which brachial artery blood pressure measurements were obtained in duplicate from the right arm using an automated oscillometric device (BpTRU model BPM-100, BpTRU Medical Devices; Coquitlam, BC, Canada).

### Echocardiography Assessment

All LV images were collected in the left lateral decubitus position with two-dimensional echocardiography and Doppler acquisition by a single investigator and using a dedicated ultrasound (Vivid Q; GE Healthcare, Horten, Norway). The system employed a M4S Matrix Sector Array Probe (2–5 MHz), and all images were analyzed offline by a single investigator using dedicated software (EchoPAC; GE Healthcare, Horten, Norway) according to the recommendations of the American Society for Echocardiography (Quinones et al., 2002; Lang et al., 2005; Mor-Avi et al., 2011). All LV indices were determined from the average of three cardiac cycles. LV dimensions at end-diastole and end-systole were measured from parasternal long axis views. Relative wall thickness was calculated as [(2 × end-diastolic posterior wall thickness)/end-diastolic LV internal diameter], while LV mass was calculated according to the area-length formula (Lang et al., 2005) and indexed to body surface area. End-diastolic and systolic volumes and ejection fractions were derived from Simpson’s biplane analysis of apical four and two-chamber views. Stroke volume was calculated from LV outflow tract diameters and velocity time integrals and multiplied by heart rate to determine cardiac output. To control for the potential differences in body size between athlete groups, end-diastolic volume, stroke volume and cardiac output were indexed to body surface area. Pulsed-wave Doppler at the tips of the mitral valve leaflet was used to determine peak early (E) and late (A) transmitral filling velocities, and the resultant E/A ratio. Pulsed-wave tissue Doppler imaging was performed at the septal and lateral mitral annulus and averaged to determine isovolumetric relaxation time and peak early (E’), late (A’), and systolic (S’) mitral annular velocities.

Parasternal short axis images at the level of the mitral valve (basal) and apex (apical) were analyzed using speckle-tracking software in accordance with current guidelines (Mor-Avi et al., 2011). Raw traces were imported into customized post-processing software (2D Strain Analysis Tool, Stuttgart, Germany), which normalizes the temporal sequence of heart rate by interpolating the data into 600 points in systole and 600 points in diastole using a standard cubic spline algorithm. Basal and apical peak rotation and rotation rate in systole and diastole were determined. Twist was determined as the maximum value obtained when subtracting the frame-by-frame basal rotation from the frame-by-frame apical rotation, while torsion was calculated by dividing twist by LV length. Peak systolic twisting rate, early diastolic untwisting rate and time to peak twisting/untwisting rates were derived in the same manner from frame-by-frame basal and apical rotation rate data.

### Statistical Analysis

Statistical analyses were performed using Statistical Package for Social Science software (IBM Corporation, Armonk, NY, United States) and GraphPad Prism (GraphPad Software Inc., La Jolla, CA, United States). Data were assessed for normality using Shapiro-Wilk tests and Q–Q plot analyses. Between group differences were assessed using independent *t*-tests and Mann-Whitney *U*-tests for normally and non-normally distributed data. Given a significant difference in supine heart rates between groups, early diastolic indices (E and E’), which are influenced by heart rate, were also compared using an analysis of covariance with heart rate as the covariate. Data are presented as mean ± SD unless otherwise noted, with *P* < 0.05 considered statistically significant.

## Results

Athlete characteristics are presented in Table 1. For runners, sprint/power athletes were defined as those who competed in 100–400 m races and high jump, while distance athletes competed in races ≥800 m. Short distance swimmers comprised those competing in 50–200 m races while middle-distance/distance athletes competed in 400–1500 m races. Eleven swimmers swam freestyle, 3 swam breaststroke and 2 swam backstroke. Groups were predominantly male (81%) and white (69%) and had similar body dimensions. Swimmers participated in competitive training for a longer duration and engaged in a larger volume of weekly training, but within the swimmer and runner groups, there was no difference in these variables between distance and sprint athletes. Supine blood pressures were similar between groups, but heart rate was higher in swimmers. LV indices are presented in Table 2. Cardiac output and cardiac output index were larger in swimmers, while all other global LV systolic function indices and systolic mechanics were similar between groups. For global LV diastolic function, early diastolic indices were higher in runners including E and E’, as well as the E/A ratio. When heart rate was entered as a covariate, E’ remained different between groups (*p* = 0.01) while E was trending (*p* = 0.06). For diastolic mechanics, runners had a shorter time to peak untwisting rate and a lower apical rotation rate in diastole compared to swimmers. LV structure was similar between groups.

**Table 1:** Athlete characteristics.

Variable	Runners	Swimmers	*P-*value
Age (year)	23 (2)	22 (3)	0.22
Sex: male, female (no)	13, 3	13, 3	–
Race: black, white (no)	5, 11	5, 11	–
Height (m)	1.86 (0.13)	1.80 (0.07)	0.12
Mass (kg)	71.3 (9.3)	74.0 (9.0)	0.41
Body mass index (kg⋅m^-2^)	22.6 (2.0)	22.9 (1.7)	0.60
Body surface area (m^2^)	1.87 (0.16)	1.92 (0.15)	0.41
Supine systolic BP (mmHg)	95 (9)	100 (10)	0.14
Supine diastolic BP (mmHg)	54 (8)	55 (7)	0.76
Supine heart rate (bpm)	49 (6)	56 (5)	**0.001**
*Training history*			
Competitive training duration (year)	8 (3)	12 (4)	**0.02**
Average training volume (h⋅wk^-1^)	17 (5)	23 (8)	**0.01**
Sport			
Run: sprint/power	10	0	–
Run: distance	6	0	–
Swim: sprint	0	6	–
Swim: middle distance/distance	0	10	–

*Data are mean (SD). BP, blood pressure. Bolded values in *P*-value column indicate significant between-group differences.*

**Table 2:** Indices of left ventricular structure, global function, and mechanics.

Variable	Runners	Swimmers	*P-*Value
**LEFT VENTRICLE (LV) STRUCTURE**			
LV internal diameter – diastole (cm)	5.3 (0.4)	5.2 (0.4)	0.37
LV internal diameter – systole (cm)	3.6 (0.3)	3.6 (0.4)	0.96
LV length – diastole (cm)	8.4 (0.6)	8.6 (0.8)	0.33
Relative wall thickness	0.33 (0.04)	0.34 (0.04)	0.22
LV mass index (g ⋅ m^-2^)	133.4 (23.7)	129.2 (25.8)	0.64
End-diastolic volume (ml)	126 (16)	137 (26)	0.06
End-diastolic volume index (ml ⋅ m^-2^)	67 (8)	71 (11)	0.29
End-systolic volume (ml)	50 (7)	57 (11)	0.32
**GLOBAL LV SYSTOLIC FUNCTION**			
Stroke volume (ml)	94 (22)	99 (25)	0.55
Stroke volume index (ml⋅ m^-2^)	50 (11)	51 (11)	0.75
Cardiac output (L ⋅ min^-1^)	4.7 (1.2)	5.8 (1.5)	**0**.**04**
Cardiac output index (L⋅ min^-1^ ⋅ m^-2^)	2.5 (0.6)	3.0 (0.7)	**0.04**
Ejection fraction (%)	60 (2)	58 (2)	0.08
S’ mean (cm ⋅ s^-1^)	9 (2)	9 (2)	0.44
**GLOBAL LV DIASTOLIC FUNCTION**			
E (cm ⋅ s^-1^)	87 (11)	76 (13)	**0.02**
A (cm ⋅ s^-1^)	28 (8)	29 (5)	0.12
E/A ratio	3.29 (0.72)	2.68 (0.59)	**0**.**005**
E’ mean (cm ⋅ s^-1^)	18 (2)	16 (2)	**0**.**01**
A’ mean (cm ⋅ s^-1^)	6 (1)	6 (1)	0.69
Isovolumetric relaxation time (ms)	74 (11)	73 (12)	0.92
E/E’ mean ratio	5.0 (1.0)	4.9 (1.1)	0.69
**LV SYSTOLIC MECHANICS**			
Twist (deg)	11.9 (4.9)	11.8 (5.6)	0.98
Torsion (deg ⋅ cm^-1^)	1.4 (0.6)	1.3 (0.6)	0.75
Twisting rate (deg ⋅ s^-1^)	70.6 (19.4)	80.2 (29.8)	0.29
Time to peak twisting rate (% systole)	49 (8)	49 (9)	0.81
Basal rotation (deg)	-4.8 (2.9)	-5.5 (2.9)	0.52
Basal rotation rate – systole (deg ⋅ s^-1^)	-45.9 (16.1)	-55.1 (15.7)	0.12
Apical rotation (deg)	7.4 (2.3)	7.3 (4.1)	0.89
Apical rotation rate – systole (deg ⋅ s^-1^)	45.4 (20.1)	58.6 (24.2)	0.12
**LV DIASTOLIC MECHANICS**			
Untwisting rate (deg ⋅ s^-1^)	-68.4 (30.3)	-76.9 (27.5)	0.42
Time to peak untwisting rate (% diastole)	5 (4)	12 (10)	**0**.**01**
Basal rotation rate – diastole (deg ⋅ s^-1^)	35.3 (17.6)	35.6 (18.2)	0.95
Apical rotation rate – diastole (deg ⋅ s^-1^)	-40.4 (15.4)	-56.8 (23.7)	**0**.**03**

*Data are mean (SD). A, peak late transmitral filling velocity. A’ mean, mean mitral annular peak late velocity. E, peak early transmitral filling velocity. E’ mean, mean mitral annular peak early velocity. S’ mean, mean mitral annular peak systolic velocity. Bolded values in *P*-value column indicate significant between-group differences.*

## Discussion

In a cohort of elite-level athletes, we demonstrate increased early diastolic filling in runners compared to swimmers, while all other indices of LV dimensions and systolic function/mechanics were similar between groups. This improved early diastolic filling in elite runners may be attributed to enhanced LV diastolic mechanics, increased preload due to greater blood volume (which we did not specifically examine), or a combination of both. The observations from this study suggest the exercise stimulus of running vs. swimming may be capable of producing distinct adaptations in LV diastolic, but not systolic, function.

While presently debated, there is evidence to suggest an association between aerobic exercise training and enhanced early diastolic filling, characterized using both preload-dependent transmitral filling velocity, and mitral annular velocity measurements which rely less on preload (George and Somauroo, 2012). Cross-sectional comparisons have observed enhanced early diastolic filling indices in swimmers relative to sedentary individuals (Caso et al., 2002; Venckunas et al., 2008a; Santoro et al., 2015), while only one study has examined differences between elite long and middle-distance runners and swimmers and observed no difference in transmitral filling velocities between groups (Venckunas et al., 2008a). We observed higher early transmitral filling and mitral annular velocities and a higher E/A ratio in our runner cohort, which remained significant or trending when controlling for heart rate, suggesting runners have enhanced early diastolic function relative to swimmers. While the untwisting rate was similar between groups, the time to peak untwisting rate occurred earlier in the diastole in runners. LV untwisting is an important component of early diastole as it precedes diastolic suction and subsequent filling (Burns et al., 2009). Thus, the capacity to reach peak untwisting faster could prolong the early filling phase leading to increased values of early diastolic filling. No swim training studies have examined LV mechanics, but land-based aerobic exercise training in young adults has been shown to enhance untwisting mechanics (Weiner et al., 2015). The shorter time to peak untwist may also explain the observation of attenuated diastolic apical rotation rates in runners relative to swimmers. In particular, the apex may not need to untwist as much given it reaches peak untwisting sooner. The differences in diastolic apical rotation rate may also be partially explained by the differences in heart rate.

The enhanced early diastolic function observed in runners suggests this land-based exercise modality may provide a greater physiological stimulus for exercise-induced cardiac adaptation. The elevated E and E/A ratio in runners, which are both preload dependent measures, could also be attributed to a greater blood volume. Upright aerobic exercise training has been shown to elicit significant increases in blood volume in young males compared to supine aerobic exercise training (Ray et al., 1990). Additionally, female runners have been shown to have a greater blood volume than female swimmers (Parker Jones et al., 1999). Thus, the observed sport-specific improvements in diastolic function may be related to adaptations in the LV, blood volume, or both. Self-reported training history and weekly training volume were both lower in runners; thus, despite training for fewer years and fewer hours per week they still demonstrated increased resting diastolic function.

It is also possible that the diastolic function observed in runners was a necessary adaptation for their exercise modality, rather than a greater adaptation. Investigations on land have demonstrated that the supine posture promotes venous return, increasing preload, early diastolic filling and subsequently stroke volume (Sato et al., 1999; Warburton et al., 2002). Additionally, earlier work in young men demonstrated increased heart volume using x-ray following head-up immersion in water in comparison to upright and supine land postures (Lange et al., 1974). Thus, the combined supine and immersed position of swimming should function to promote diastolic filling. Previous studies have observed swimming and running at a similar oxygen uptake level have elicited similar cardiac outputs but lower heart rates during swimming, suggesting an increased stroke volume (Holmer, 1979). Thus, it is possible that swimmers do not need to further augment diastolic function to the level of a runner, because the position and water immersion aspects of their exercise modality would promote filling. While we did not compare our athletes to a sedentary control group, we would consider both groups to exhibit exercise-induced LV remodeling as they represent athletes at the elite level of their respective sports, and their values are generally higher than age-matched reference values (Lang et al., 2005; Nagueh et al., 2009). For example, the average E/A ratio is 1.88 and 1.53 for 16–20 and 21–40 year olds, respectively (Nagueh et al., 2009). This highlights that the attenuated diastolic values in swimmers relative to runners should not be interpreted as reduced diastolic function, but on a continuum of adaptations to exercise training.

The only difference in systolic function between groups was cardiac output, which can be attributed to higher heart rates in swimmers given stroke volumes were similar. Resting bradycardia with aerobic exercise training is well characterized, and while some have observed no difference in resting heart rate between aerobic sport modalities (Colan et al., 1987; Wasfy et al., 2015), higher resting heart rates have been observed in swimmers compared to runners (Nualnim et al., 2011). For LV dimensions, cross-sectional comparisons of swimmers to sedentary controls suggest swimmers experience exercise-induced LV remodeling including increases in wall thickness, internal diastolic diameters and LV mass index (Colan et al., 1987; Caso et al., 2002; Venckunas et al., 2008a; Santoro et al., 2015). Comparisons between swimmers and runners have generally demonstrated no difference in LV internal diastolic diameter or LV mass index (Colan et al., 1987; Venckunas et al., 2008a), which is in agreement with our observations. Colan et al. (1987) argued that swimmers experience an intermediate level of LV remodeling that would fall on the spectrum between running and powerlifting athletes, and that their degree of adaptation is attributed to both the volume overload typically associated with endurance (i.e., isotonic) training and the pressure overload typically associated with resistance (i.e., isometric) training. This was based on their observation that end-systolic wall thickness in swimmers was larger than runners but smaller than power lifters. The long-standing view that isometric-based training elicits concentric remodeling, however, has recently been discredited (Spence et al., 2011). We observed no difference in relative wall thickness, which suggests both sports, regardless of their isotonic and isometric components, provide a similar physiological stimulus for remodeling.

The strengths of our study include the assessment of elite athletes during their competition season and our comprehensive assessment of LV function including both traditional measures of global function and more advanced speckle-tracking assessments of systolic and diastolic mechanics. However, we do acknowledge several limitations. Each group contained a heterogeneous mixture of sex, race, and sport distance, all of which can influence LV adaptations with exercise training (Sharma, 2003). We matched athletes in each group based on sex and race; therefore, the potential confounding effects of these factors should be minimized. Differences in LV structure have been documented between sprint and distance runners; however, there were no differences in diastolic parameters (Venckunas et al., 2008b). Within swimming, one study documented differences in LV structure and the E/A ratio between endurance (i.e., 400–800 m) and “strength” (i.e., 50–100 m) swimmers (D’Andrea et al., 2003). Strength swimmers also performed 2 h per day of land-based strength training in comparison to 1 h per week in the endurance group; therefore it is difficult to ascertain whether these differences were exclusively attributed to the swim training. Within our swimmer cohort, we are limited in our ability to compare LV parameters between sport distances given race distributions (i.e., 71% of sprint swimmers were black vs. 10% of middle distance/distance swimmers). However, comparison of diastolic indices between sprint and middle distance/distance swimmers, both with and without race as a covariate, revealed no significant differences between distance groups. We are also unable to discriminate the amount of training spent performing isotonic and isometric activities. Given the elite status of all athletes, it is safe to assume they were engaging in the best training practices for their respective event and therefore represent high-performance running and swimming athletes. For a more comprehensive understanding of cardiac differences between runners and swimmers, future investigations should consider examining the right ventricle and atria, as well as longitudinally tracking changes with training in order to draw more definitive conclusions regarding sport-specific adaptations. Furthermore, the practical implications of enhanced resting diastolic function in runners compared to swimmers is unknown. Thus, future studies should consider comparing LV performance during both supine and upright exercise in these athlete groups.

## Conclusion

Our cross-sectional comparison of elite swimmers and runners suggest running is associated with faster LV untwisting during diastole and enhanced early diastolic filling. Further research is required to delineate whether these observations are a product of a superior exercise stimulus, or a necessary adaptation to promote filling during upright exercise. The different exercise stimuli of swimming and running did not appear to affect LV structure or systolic function, as these indices were similar between groups. Overall, our findings lend further support to the concept of sport-specific differences in LV function.

## Author Contributions

KC, AC, JS, RA, AW, and JB were involved in data collection. KC performed the data and statistical analysis. KC, PM, and JB interpreted the data. KC drafted the manuscript and revisions were done by KC, PM, and JB. All authors were involved in the conception and design of the study and approved the final version of the manuscript.

## Conflict of Interest Statement

The authors declare that the research was conducted in the absence of any commercial or financial relationships that could be construed as a potential conflict of interest.
